# Genome-wide association study of behavioural and psychiatric features in human prion disease

**DOI:** 10.1038/tp.2015.42

**Published:** 2015-04-21

**Authors:** A G B Thompson, J Uphill, J Lowe, M-C Porter, A Lukic, C Carswell, P Rudge, A MacKay, J Collinge, S Mead

**Affiliations:** 1 MRC Prion Unit, Department of Neurodegenerative Disease, University College London (UCL) Institute of Neurology, London, UK; 2 NHS National Prion Clinic, National Hospital for Neurology and Neurosurgery, UCL Hospitals NHS Foundation Trust, London, UK; 3 NHS Highland Mental Health Services, Argyll and Bute Hospital, Lochgilphead, Argyll, UK

## Abstract

Prion diseases are rare neurodegenerative conditions causing highly variable clinical syndromes, which often include prominent neuropsychiatric symptoms. We have recently carried out a clinical study of behavioural and psychiatric symptoms in a large prospective cohort of patients with prion disease in the United Kingdom, allowing us to operationalise specific behavioural/psychiatric phenotypes as traits in human prion disease. Here, we report exploratory genome-wide association analysis on 170 of these patients and 5200 UK controls, looking for single-nucleotide polymorphisms (SNPs) associated with three behavioural/psychiatric phenotypes in the context of prion disease. We also specifically examined a selection of candidate SNPs that have shown genome-wide association with psychiatric conditions in previously published studies, and the codon 129 polymorphism of the prion protein gene, which is known to modify various aspects of the phenotype of prion disease. No SNPs reached genome-wide significance, and there was no evidence of altered burden of known psychiatric risk alleles in relevant prion cases. SNPs showing suggestive evidence of association (*P*<10^−5^) included several lying near genes previously implicated in association studies of other psychiatric and neurodegenerative diseases. These include *ANK3*, *SORL1* and a region of chromosome 6p containing several genes implicated in schizophrenia and bipolar disorder. We would encourage others to acquire phenotype data in independent cohorts of patients with prion disease as well as other neurodegenerative and neuropsychiatric conditions, to allow meta-analysis that may shed clearer light on the biological basis of these complex disease manifestations, and the diseases themselves.

## Introduction

The human prion diseases are a group of rare neurodegenerative conditions that occur in sporadic, inherited and acquired forms.^1^ Their clinical manifestations are highly variable both within and between these different aetiological types, and often include prominent neuropsychiatric symptomatology.^2, 3^ The causes of this clinical heterogeneity are incompletely understood. Some modifiers of the clinical phenotype are well established, such as the polymorphic genotype at codon 129 of the prion protein gene (*PRNP*), but known factors only account for a small minority of the total variability seen.^4, 5, 6^

Clinical study of these diseases is challenging because of their rarity and their typically rapid progression, which makes detailed prospective follow-up difficult to achieve. Psychiatric symptoms are particularly difficult to study as patients often have substantial cognitive impairment with limited expressive language function by the time the diagnosis is made, so they are unable to describe their internal experiences to allow their symptoms to be characterized. Further, psychiatric manifestations are often intense but transient.^7^

We recently undertook a large clinical study of behavioural disturbance and psychiatric symptoms (BPS) in human prion disease in the context of the National Prion Monitoring Cohort (‘the Cohort'), an ongoing prospective natural history study that has aimed to recruit all patients with all types of prion disease in the United Kingdom since October 2008. This used clinical data from more than 300 patients enrolled in the Cohort and/or the preceding PRION-1 clinical trial to characterize in as much detail as possible the range of BPS seen in prion disease, as well as their prevalence, natural history and observed response to symptomatic treatments. The clinical aspects of this work have now been published.^7^ Full methodological details of the PRION-1 trial and the Cohort study have been published previously.^8, 9^

A large number of patients included in these clinical studies were included in a genome-wide association study (GWAS) of susceptibility to prion disease,^10^ and therefore have single-nucleotide polymorphism (SNP) genotype data publicly available. This GWAS found that several SNPs at the *PRNP* locus itself were very strongly associated with all types of prion disease, with this association being driven by linkage disequilibrium with the polymorphism at codon 129 (which was itself one of the genotyped SNPs). No other SNPs reached genome-wide significance, and no SNPs that had previously been found to show genome-wide association with other neurodegenerative diseases showed any association with prion disease.

The detailed clinical data regarding the presence or absence of BPS established for our recent clinical study allows us to operationalise specific behavioural/psychiatric phenotypes as traits in human prion disease, and thereby presents the opportunity to carry out a GWAS looking for genetic modifiers of these traits. A similar approach has previously been taken in other neurodegenerative diseases that may cause psychiatric symptoms, such as Alzheimer's disease,^11^ and this approach has the potential to provide valuable clues to the molecular pathways that may underlie these complex disease manifestations.

## Materials and methods

All patients were referred to the NHS National Prion Clinic, and were enrolled in the PRION-1 trial and/or the National Prion Monitoring Cohort. Uptake of enrolment in these clinical research studies is extremely high (>95% in the Cohort), so they provide a highly representative sample of patients seen in this clinical setting. Patients were diagnosed with probable sporadic Creutzfeldt-Jakob disease (CJD) according to World Health Organisation criteria with the addition of brain MRI as a supportive investigation as recommended by the MRI-CJD consortium.^12^ Variant CJD was diagnosed using established criteria.^13^ Patients were diagnosed with inherited prion disease if *PRNP* genotyping showed the presence of a pathogenic mutation in the presence of a consistent clinical syndrome. All patients included in this study underwent diagnostic genotyping to confirm or rule out the presence of a pathogenic *PRNP* mutation. Patients were diagnosed with iatrogenic CJD using sporadic CJD criteria in the presence of a history of relevant exposure (for example, to implicated cadaveric human growth hormone). Approximately 2/3 of patients had neuropathological examination of brain tissue (post mortem or from biopsy), or tonsil biopsy (for variant CJD), which was used to confirm the diagnosis.

### Clinical data from PRION-1 and the Cohort

PRION-1 was an open-label, patient preference trial of quinacrine for all types of human prion disease, which recruited patients from 2001 to 2008. The trial showed no effect of quinacrine on survival or any of the rating scales used as secondary outcome measures.^8, 14^ The National Prion Monitoring Cohort is an ongoing natural history study of all types of prion disease, which has been enrolling patients since October 2008.^9^

In both studies, patients were enrolled and followed up throughout their disease course whenever possible, with clinical data recorded by a neurologist at each assessment. For the analysis below, three particular sets of data were used to identify patients with BPS:

First symptoms: at enrolment, all patients and/or carers were asked to recall the first symptom that had been noticed or reported when the illness began. If more than one symptom was felt to have appeared together these were all included. It was specifically recorded whether behavioural and psychiatric symptoms were among these first symptoms for all patients.

Symptoms at time of assessment: the presence and severity/frequency of a range of specific symptoms were recorded at each assessment. These included ‘hallucinations' and ‘depressive symptoms'.

Indications for drug prescription: details of all drugs prescribed during the period of study were recorded, including indication for treatment.

The clinical case notes of selected patients identified from the research data, including all of those prescribed medication for BPS, were reviewed in detail by one of the authors (AGBT) to confirm the presence and nature of the clinical features.

These sources of clinical information were combined to classify each patient on the basis of three specific behavioural/psychiatric phenotypes:

- Presence of any behavioural disturbance or psychiatric symptoms at the onset of the illness (that is, among the first symptoms).
- Presence of psychotic features (hallucinations and delusions) at any stage of the illness.
- Presence of mood disorder (including low mood, emotional lability, apathy and withdrawal) at any stage of the illness.

Genome-wide association analysis was performed on 170 cases (114 sporadic CJD, 33 inherited prion disease, 22 variant CJD and 1 iatrogenic CJD) and 5200 UK controls provided by the Wellcome Trust Case Control Consortium. Controls were genotyped on the Illumina 1.2 M Custom Duo array; cases on the Illumina 660 K array (Illumina, San Diego, CA, USA). Analyses were carried out comparing (1) cases with each phenotype (prion+) with cases without the phenotype (prion–), and (2) prion+ cases with controls (that is, total of six analyses).

All of these individuals had been genotyped previously for inclusion in a GWAS of susceptibility to prion disease,^10^ and full details of patient and control samples, genotyping and quality control are included in that paper. In the current study, Fisher's exact test was used for the association analyses because of the relatively small number of patients. 518 938 SNPs were included in the analysis after quality control. Ethnic outliers detected using a multidimensional scaling plot were excluded. Data manipulation and statistical analysis were carried out using PLINK (http://pngu.mgh.harvard.edu/purcell/plink/).

The rationale for including a comparison of prion+ cases with controls was that if a hypothetical risk allele existed for a psychiatric phenotype in the context of prion disease, then it would be expected to be most common in prion+ cases, followed by controls (who may include individuals that would be at risk if they were to develop prion disease), followed by prion− cases (who have not developed the phenotype despite having prion disease). The comparison with controls would therefore produce an odds ratio (OR) closer to 1, but nevertheless might be more likely to show a high level of statistical significance because of the much larger numbers of individuals included, whereas the comparison with prion− cases would be expected to produce a larger OR but might fail to reach such high levels of statistical significance. As all of these individuals were previously included in the GWAS of susceptibility to prion disease,^10^ which showed no strong associations apart from SNPs at the *PRNP* locus itself, it would not be expected that association with prion disease itself would lead to spurious strong associations in the prion+ vs controls analysis, and if this was the case then the same SNP would not be expected to show any association in the prion+ vs prion− analysis.

We also planned to specifically examine a small selection of candidate SNPs in a hypothesis-driven manner, in light of the limited statistical power that could be achieved in the genome-wide analysis, given the relatively small number of cases. We reviewed previously published GWASs in psychiatric conditions characterised by psychosis and/or mood disorder (schizophrenia,^15^ bipolar affective disorder^16, 17, 18^ and major depressive disorder^19^ to identify a list of candidate SNPs that have shown association with these conditions at genome-level significance, on the basis that these might also show association with the behavioural/psychiatric phenotypes in prion disease. These SNPs are listed in Table 1. We also included the codon 129 polymorphism of *PRNP* (SNP rs1799990), as this is known to modify other aspects of the phenotype of prion disease (for example, rate of disease progression, and incubation time in acquired prion disease) and also to confer susceptibility to prion disease (as shown in the GWAS for prion disease mentioned above^10^). There was no evidence of population structure in the United Kingdom and no corrections were made.

## Results

Table 2 shows details of the SNPs with the most significant associations in each analysis. No SNPs reached the standard threshold for GWASs (*P*<5 × 10^−8^). All data are publicly available.

The single most significant association was in the prion+ vs controls analysis for psychiatric symptoms at onset, for a SNP (rs10509125) lying within the Ankyrin 3 (*ANK3*) gene (*P*=1.43 × 10^−6^, OR=2.09) (see Table 2). In the prion+ vs prion− analysis, association for this SNP was less significant but effect size was greater (*P*=3.5 × 10^−5^, OR=2.52).

A cluster of three SNPs on chromosome 6p were most strongly associated with the presence of psychotic features in the prion+ vs controls analysis (rs1055569; *P*=2.66 × 10^−6^, OR=2.36), and also showed association in the prion+ vs prion– analysis (*P*=1.08 × 10^−4^, OR=2.49). These SNPs span the 5′ end of the non-protein-coding human leukocyte antigen complex group 26 (*HCG26*) gene.

The most significant association in the mood disorder analyses was in the prion+ vs controls analysis for a SNP lying within the acylglycerol kinase (*AGK*) gene (rs7789850; *P*=2.16 × 10^−6^, OR=4.6). *AGK* encodes a mitochondrial membrane protein involved in lipid and glycerolipid metabolism.

Closely associated SNPs rs1219407 and rs478903, which showed the strongest association with mood disorder in the prion+ vs prion− analysis, lie about 200 kb from the *SORL1* gene. rs6867820, another SNP that showed association with mood disorder in our analyses, lies 55 kb from the SNCAIP gene, which encodes Synphilin-1.

Table 1 summarises the association results for the candidate SNPs identified from previously published GWAS in psychiatric conditions (schizophrenia,^15^ bipolar affective disorder^16, 17, 18^ and major depressive disorder^19^ and in prion disease).^10^ The SNP implicated by the prion disease GWAS is *PRNP* codon 129. As this SNP is known to be strongly associated with prion disease, it is not meaningful to include results for the prion+ vs controls analysis (as expected these showed strong association). None of the candidate SNPs identified from GWAS in psychiatric conditions showed evidence of association with the behavioural/psychiatric phenotypes in prion disease in our analysis, in light of the number of tests being performed. In addition, we performed an analysis of the burden of risk alleles for psychiatric conditions (9 GWA loci shown in Table 1 *P*<5x10^−8^) by the score method using PLINK. We weighted allele contributions based on the logarithm of OR in the discovery study. There were no statistically significant differences between mean scores for prion+ vs prion− or prion+ vs controls (six tests, all *P*>0.1).

## Discussion

We have performed exploratory candidate SNP, risk allele burden and genome-wide association studies to look for evidence of genetic loci associated with three behavioural/psychiatric phenotypes in the context of prion disease. As the number of patients included is small, these studies are only powered to detect very strongly associated SNPs, and the lack of any reaching genome-wide significance certainly does not rule out the possibility of major genetic modifiers of these phenotypes. Patients with all prion disease types were considered together in this analysis, so it is possible that aetiology-specific associations may have been missed or underestimated. The main aims of the study were to establish feasibility of this approach and to test for a broad role of a limited number of definite genetic risk factors discovered in primary psychiatric disease.

Hypotheses are proposed here for follow-up in other cohorts of prion disease or related neurodegenerative diseases.^11^ Reviewing the most strongly associated SNPs from our study we identified several loci that may be of interest, as they have previously been implicated in genetic studies of psychiatric or other neurodegenerative disorders.

The strongest evidence of association was for a SNP lying within the *ANK3* gene, in the analysis of psychiatric features at onset. There is strong evidence for association of other SNPs within *ANK3* with bipolar disorder from a large collaborative GWAS,^16^ although the genotyped SNPs reported in that study showed no evidence of association in our analysis (see Table 1). Ankyrin 3 is thought to participate in the maintenance/targeting of ion channels and cell adhesion molecules at the nodes of Ranvier and initial axon segments.^20^

The SNPs on chromosome 6 that we found to be trending towards association with psychotic features in prion disease lie within a chromosomal region (6p21.3–22.1) that has been implicated in genetic studies of susceptibility to both schizophrenia and bipolar disorder, without a single locus emerging as the dominant source of association.^15, 21^ It is possible that this apparent inconsistency is related to the unusual patterns of recombination around the major histocompatibility complex, which also lies in this region.^22^ Four of the top five candidate genes for schizophrenia risk listed on SzGene.org lie within this region (www.szgene.org). Although we must be conservative in our conclusions because of the small patient numbers and the failure of any SNPs to reach genome-wide significance, this suggestion of an overlap with the genetics of ‘primary' psychiatric disorders is intriguing.

Large GWASs using both SNPs and copy number variants have previously identified genetic loci with an effect across multiple psychiatric and neurodevelopmental conditions, with the overlap between risk loci for schizophrenia and bipolar disorder being particularly well established.^23, 24, 25, 26, 27^ This has been interpreted as evidence that there are genetic risk factors for psychosis that are not disease specific.^25^ It is conceivable that a genetic factor conferring susceptibility to schizophrenia or bipolar disorder might also increase the likelihood of an individual developing psychotic features in the context of neurodegenerative disease. Our results although negative, provide an intriguing hint that this may be the case in prion disease, and we wish to encourage further investigation of this hypothesis.

## Figures and Tables

**Table 1:** Summary of association results from our analysis for candidate single-nucleotide polymorphisms identified from previously published genome-wide association studies of psychiatric conditions (schizophrenia, bipolar affective disorder and major depressive disorder) and prion disease

*Candidate SNPs identified from other GWAS*							*Psychotic symptoms*						*Mood disorder*						*BPS at onset*					
							*Prion+ vs controls*			*Prion+ vs prion*−			*Prion+ vs controls*			*Prion+ vs prion*−			*Prion+ vs controls*			*Prion+ vs prion*−		
*SNP*	*Chr*	*BP*	*MAF*	*Closest gene (ref seq)*	*Identified in GWAS for…*	*Reference*	*GWAS* P*-value*	*OR*	*95% CI*	*GWAS* P*-value*	*OR*	*95% CI*	*GWAS* P*-value*	*OR*	*95% CI*	*GWAS* P*-value*	*OR*	*95% CI*	*GWAS* P*-value*	*OR*	*95% CI*	*GWAS* P*-value*	*OR*	*95% CI*
*SNPs implicated in GWAS for psychiatric conditions*																								
rs6932590	6	27356910	0.27	*PRSS16*	Schizophrenia	Stefansson *et al.*^15^	0.42	0.82	0.54–1.24	0.90	0.96	0.57–1.61	0.26	0.79	0.53–1.16	0.70	0.89	0.53–1.47	0.73	0.93	0.66–1.30	0.44	1.23	0.74–2.03
rs3131296	6	32280971	0.15	*NOTCH4*	Schizophrenia	Stefansson *et al.*^15^	0.01	0.43	0.22–0.85	0.44	0.71	0.31–1.60	0.01	0.49	0.27–0.89	0.71	0.85	0.40–1.83	2.09E−03	0.44	0.25–0.77	0.34	0.67	0.32–1.43
rs9960767	18	51306000	0.05	*TCF4*	Schizophrenia	Stefansson *et al.*^15^	0.69	0.75	0.31–1.85	0.46	0.59	0.21–1.68	1.00	0.91	0.42–1.95	0.64	0.74	0.29–1.94	1.00	0.97	0.49–1.91	0.81	0.81	0.32–2.05
rs12807809	11	124111495	0.17	*NRGN*	Schizophrenia	Stefansson *et al.*^15^	0.23	1.3	0.84–2.00	0.67	1.17	0.67–2.03	0.01	1.66	1.14–2.42	0.02	1.95	1.13–3.37	1.00	0.97	0.65–1.45	0.17	0.68	0.40–1.17
rs13211507	6	28365356	0.12	*PGBD1*	Schizophrenia	Stefansson *et al.*^15^	0.10	0.56	0.28–1.11	0.44	0.71	0.31–1.60	0.16	0.65	0.36–1.17	0.71	0.85	0.40–1.83	0.64	0.87	0.54–1.41	0.25	1.64	0.76–3.56
rs6913660	6	27199404	0.19	*HIST1H2BJ*	Schizophrenia	Stefansson *et al.*^15^	0.21	0.7	0.43–1.16	0.19	0.66	0.36–1.21	0.68	0.9	0.59–1.38	0.89	0.96	0.55–1.67	0.77	0.92	0.63–1.36	1.00	1.01	0.58–1.75
rs1938526	10	61970389	0.06	*ANK3*	BPAD	Ferreira *et al.*^16^	0.85	1.02	0.50–2.10	0.53	0.74	0.31–1.75	0.73	1.09	0.57–2.09	0.68	0.8	0.35–1.81	0.35	1.29	0.74–2.23	1.00	1.07	0.48–2.38
rs1064395	19	19222735	0.16	*NCAN*	BPAD	Cichon *et al.*^17^	0.32	1.26	0.80–1.97	0.46	1.25	0.70–2.22	0.31	1.25	0.82–1.89	0.47	1.26	0.72–2.22	0.18	1.31	0.90–1.90	0.20	1.48	0.83–2.63
rs2251219	3	52559827	0.39	*PBRM1*	BPAD/MDD	McMahon *et al.*^18^	0.20	1.26	0.88–1.79	0.30	1.29	0.82–2.01	0.31	1.2	0.86–1.66	0.44	1.22	0.79–1.88	0.10	1.29	0.95–1.73	0.10	1.46	0.95–2.26
rs2289247	3	52702297	0.41	*GNL3*	BPAD/MDD	McMahon *et al.*^18^	0.04	1.44	1.01–2.05	0.05	1.58	1.01–2.46	0.18	1.26	0.91–1.74	0.27	1.3	0.85–2.01	0.08	1.32	0.98–1.77	0.06	1.51	0.98–2.33
rs4238010	12	3988578	0.13	*CCND2*	MDD	Muglia *et al.*^19^	0.19	1.37	0.86–2.18	0.88	1.05	0.58–1.87	1.00	0.96	0.59–1.55	0.08	0.58	0.32–1.05	0.50	1.16	0.77–1.76	0.39	0.76	0.43–1.33
																								
*SNP implicated in GWAS for prion disease (PRNP codon 129)*																								
rs1799990	20	4628251	0.35	*PRNP*	Prion disease	Mead *et al.*^10^	—	—	—	0.62	0.83	0.42–1.62	—	—	—	0.33	0.68	0.35–1.32	—	—	—	0.1	0.56	0.30–1.06

Abbreviations: BP, base pair position - NCBI Build 36 (hg18); BPAD, bipolar affective disorder; BPS, behavioural disturbance or psychiatric symptoms; Chr, chromosome; CI, confidence interval; GWAS, genome-wide association study; MAF, minor allele frequency; MDD, major depressive disorder; OR, odds ratio for minor allele; prion+, prion disease cases with the phenotype; prion−, prion disease cases without the phenotype; SNP, single-nucleotide polymorphism.

As SNP rs1799990 (PRNP codon 129) is known to be strongly associated with prion disease, it is not meaningful to include results for prion+ vs controls analysis (as expected these showed strong association).

**Table 2:** The most strongly associated single-nucleotide polymorphisms in each of the genome-wide association analyses: prion+ vs controls and prion+ vs prion− for each of three psychiatric/behavioural phenotypes

*Phenotype*	*SNP*	*Chr*	*BP*	*MAF*	*Closest gene (ref seq)*	*Distance (kb)*	*Prion+ vs controls*			*Prion+ vs Prion-*		
							*GWAS* P*-value*	*OR*	*95% CI*	*GWAS* P*-value*	*OR*	*95% CI*
Psychotic features	rs1055569	6	31548061	0.291	HCG26	Intragenic	**2.66E−06**	**2.36**	**1.66–3.35**	1.08E−04	2.49	1.57–3.94
	rs4413654	6	31549328	0.219	HCG26	1.164	**3.69E−06**	**2.43**	**1.70–3.48**	1.02E−04	2.63	1.62–4.27
	rs2516440	6	31548476	0.294	HCG26	0.312	**4.48E−06**	**2.33**	**1.64–3.31**	1.08E−04	2.49	1.57–3.94
	rs4077732	11	11493063	0.33	GALNTL4	Intragenic	2.96E−03	0.53	0.34–0.81	**1.65E−06**	**0.3**	**0.18–0.50**
	rs7231996	18	69417499	0.131	LOC100505817	249.395	0.01	1.81	1.18–2.78	**3.14E−06**	**5.56**	**2.59–11.95**
	rs6440974	3	156553159	0.099	LOC100507537	58.976	6.56E−05	2.59	1.69–3.96	**4.47E−06**	**5.27**	**2.52–11.03**
Mood disorder	rs7789850	7	140947080	0.026	AGK	Intragenic	**2.16E−06**	**4.6**	**2.70–7.83**	2.46E−04	7.64	2.18–26.73
	rs12789145	11	94047384	0.084	PIWIL4	53.149	**3.58E−06**	**0**	**NA**	7.75E−06	0	NA
	rs6867820	5	121882424	0.224	SNCAIP	54.731	**6.03E−06**	**2.24**	**1.60–3.12**	9.74E−05	2.61	1.61–4.24
	rs1219407	11	121249388	0.073	SORL1	215.633	8.75E−05	2.6	1.68–4.02	**3.94E−06**	**7.6**	**2.83–20.39**
	rs478903	11	121190975	0.089	SORL1	181.294	1.35E−04	2.39	1.58–3.63	**6.65E−06**	**6.17**	**2.61–14.57**
	rs761998	20	14257952	0.352	FLRT3	Intragenic	0.02	0.64	0.44–0.92	**6.69E−06**	**0.35**	**0.22–0.55**
BPS at onset	rs10509125	10	61596872	0.402	ANK3	Intragenic	**1.43E**−**06**	**2.09**	**1.55–2.82**	3.50E−05	2.52	1.63–3.91
	rs561437	13	109734228	0.499	COL4A1	Intragenic	**4.77E−06**	**0.49**	**0.35–0.67**	1.15E−04	0.42	0.27–0.65
	rs7151968	14	47671186	0.205	LOC100506433	337.219	**5.83E−06**	**2.12**	**1.55–2.90**	1.60E−03	2.23	1.36–3.65
	rs4743805	9	106909655	0.165	SLC44A1	137.095	5.89E−03	1.66	1.17–2.34	**6.35E−06**	**4.51**	**2.24–9.08**
	rs9472202	6	44129264	0.208	C6orf223	47.592	3.78E−05	1.98	1.45–2.71	**8.41E−06**	**3.32**	**1.92–5.73**
	rs7040444	9	15049821	0.213	LOC389705	40.099	2.93E−03	0.52	0.33–0.81	**1.17E−05**	**0.3**	**0.17–0.52**

Abbreviations: BP, base pair position - NCBI Build 36 (hg18); BPS, behavioural disturbance or psychiatric symptoms; Chr, chromosome; CI, confidence interval; GWAS, genome-wide association study; MAF, minor allele frequency; OR, odds ratio for minor allele; prion+, prion disease cases with the phenotype; prion−, prion disease cases without the phenotype; SNP, single-nucleotide polymorphism. Bold text highlights the results from the analysis in which each SNP was amongst the 3 most significant associations.
